# From Household Chemical to Medical Emergency: Stroke Induced by Hydrogen Peroxide Ingestion

**DOI:** 10.7759/cureus.63444

**Published:** 2024-06-29

**Authors:** Krishna Padarabinda Tripathy, Saikat Banerjee, Pradip Kumar Behera, Chikkam Sandeep, Himavanth Kampli

**Affiliations:** 1 General Medicine, Kalinga Institute of Medical Sciences, Bhubaneswar, IND

**Keywords:** cerebro-vascular accident (stroke), accidental poisoning, arterial gas embolism, embolic cva, hydrogen peroxide (h2o2)

## Abstract

Hydrogen peroxide (H₂O₂) ingestion can lead to severe systemic complications, including neurological sequelae such as acute embolic stroke. We present a case of a 49-year-old male who accidentally ingested approximately 50-60 mL of 50% w/w hydrogen peroxide, resulting in encephalopathy, upper motor neuron quadriparesis, and pulmonary artery thrombosis. The patient’s altered sensorium progressed to a stupor, accompanied by acute respiratory distress and abdominal gaseous distension. Imaging revealed multifocal hypodensities in the brain and saddle thrombus in the pulmonary arteries. Hyperbaric oxygen therapy initiated after diagnosis led to a significant improvement in motor power and resolution of abdominal distension during hospitalization. The pathophysiology involves gas embolization and oxidative stress-induced thrombosis. Management includes stabilizing the patient, dilution therapy, and supportive care, with hyperbaric oxygen therapy for severe cases. Prevention strategies focus on education and proper storage. Continuous monitoring and follow-up are essential for managing hydrogen peroxide poisoning. This case underscores the need for awareness and prompt intervention in hydrogen peroxide toxicity.

## Introduction

Hydrogen peroxide (H₂O₂) is a chemical compound widely used in various industries and households due to its strong oxidizing properties. It is commonly used as a disinfectant and bleaching agent. Hydrogen peroxide is a simple peroxide composed of two hydrogen atoms and two oxygen atoms (H₂O₂). It is an odorless and colorless liquid at room temperature and is typically found in aqueous solutions of varying concentrations. Hydrogen peroxide is both a powerful oxidizer and a weak acid, decomposing into water (H₂O) and oxygen (O₂) upon breakdown: 2H2O2 → 2H2O + O2.

Hydrogen peroxide in the medical sector serves as an antiseptic for wound cleaning and is used in mouth rinses. Industrially, it is employed in the bleaching of textiles, paper, and pulp. In households, hydrogen peroxide is a common component in cleaning agents, disinfectants, and stain removers. Additionally, it is used in cosmetic applications for hair bleaching and teeth whitening.

Exposure to low concentrations (3% typical household hydrogen peroxide) usually results in mild irritation of the mouth, throat, and stomach. Common symptoms include possible vomiting, abdominal pain, and bloating. The primary risk is the formation of oxygen gas bubbles in the stomach, which can lead to gastric distension. Exposure to high concentrations (10% or higher, used in industrial or hair bleaching products) can cause severe irritation and burns to the mouth, throat, esophagus, and stomach. This often results in intense pain, vomiting (which may be bloody), and abdominal cramps. The primary risk is the development of oxygen gas embolism, where oxygen bubbles can travel to the heart or brain, leading to serious complications such as stroke, heart attack, or respiratory distress [1].

While ingestion of low concentrations of hydrogen peroxide (H₂O₂) is typically benign, higher concentrations can lead to serious toxicity, including venous or arterial gas embolism (AGE), hemorrhagic gastritis, and even death [2,3]. We report the case of a 49-year-old who accidentally ingested approximately 50-60 mL of 50% w/w hydrogen peroxide and presented with encephalopathy, features of upper motor neuron (UMN) quadriparesis, and pulmonary artery thrombosis. We suggest that the probable mechanism of tissue damage was multiple air embolisms across different vasculatures.

## Case presentation

A 49-year-old man, employed at a poultry farm, accidentally ingested approximately 50-60 mL of 50% w/w hydrogen peroxide stored in a water bottle, which serves as a sanitizing agent for cleaning pipes at his workplace. He experienced vomiting and presented to the emergency department within four hours of ingestion, exhibiting symptoms of oral irritation and a mild retrosternal burning sensation. Upon admission, his vital signs and systemic examination were normal, and he was admitted for observation. His baseline functioning was normal. Ten hours post-ingestion, the patient developed acute respiratory distress, acute abdominal gaseous distension, and altered sensorium, initially presenting as drowsiness, which progressed to stupor with a Glasgow Coma Scale score of E2V1M1, without seizures. He exhibited decreased movement in all four limbs with a bilateral plantar extensor response and no neck stiffness within a three-hour time frame. This altered state of consciousness persisted for two days with stable hemodynamics. On investigating the patient’s hemogram, liver function tests and renal function tests were normal (Table 1).

**Table 1:** Blood investigations ALP, alkaline phosphatase; GGT, gamma-glutamyl transferase; SGOT, serum glutamic oxaloacetic transaminase; SGPT, serum glutamic pyruvic transaminase

Laboratory parameter	Value	Normal range
Serum bilirubin	0.34 mg/dl	0.2-1.2 mg/dl
SGOT	37 U/L	0-40 U/L
SGPT	24 U/L	5-40 U/L
Serum ALP	60 U/L	40-129 U/L
Serum GGT	21 U/L	10-60 U/L
Serum albumin	3.0 gm/dl	3.5-5.0 gm/dl
Serum urea	33 mg/dl	12-42 mg/dl
Serum creatinine	0.53mg/dl	0.7-1.3 mg/dl
Serum sodium	141 mmol/L	136-146 mmol/L
Serum potassium	3.7 mmol/L	3.5-5.1 mmol/L
Serum calcium	7.4 mg/dl	8.6-10.3 U/L
Serum uric acid	4.6 mg/dl	3.4-7.0 mg/dl
Hemoglobin	16.1 gm/dl	13-17 gm/dl
WBC count	15,750/μL	4,000-10,000/μL
Platelet count	186,000/μL	150,000-410,000/μL

**Figure 1:**
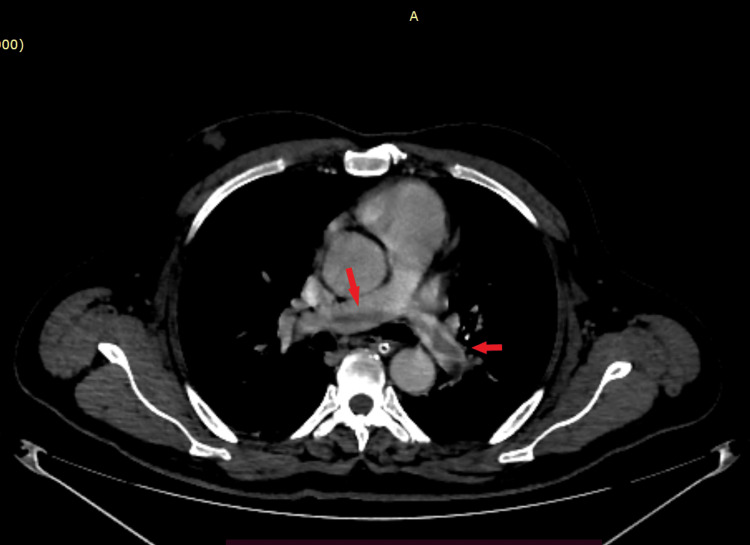
Contrast-enhanced CT thorax The red arrows indicate a saddle-shaped thrombus in the pulmonary trunk extending into the right and left pulmonary arteries.

A non-contrast CT scan of the brain was performed due to an altered sensorium, revealing ill-defined multifocal hypodensities in the bilateral high frontal and left parasagittal parietal lobes. In suspicion of embolism at other sites, screenings of the contrast-enhanced thorax and abdomen were conducted, which indicated a saddle thrombus in both pulmonary arteries extending into the segmental branches, suggestive of acute pulmonary gas embolism (Figure 1).

**Figure 2:**
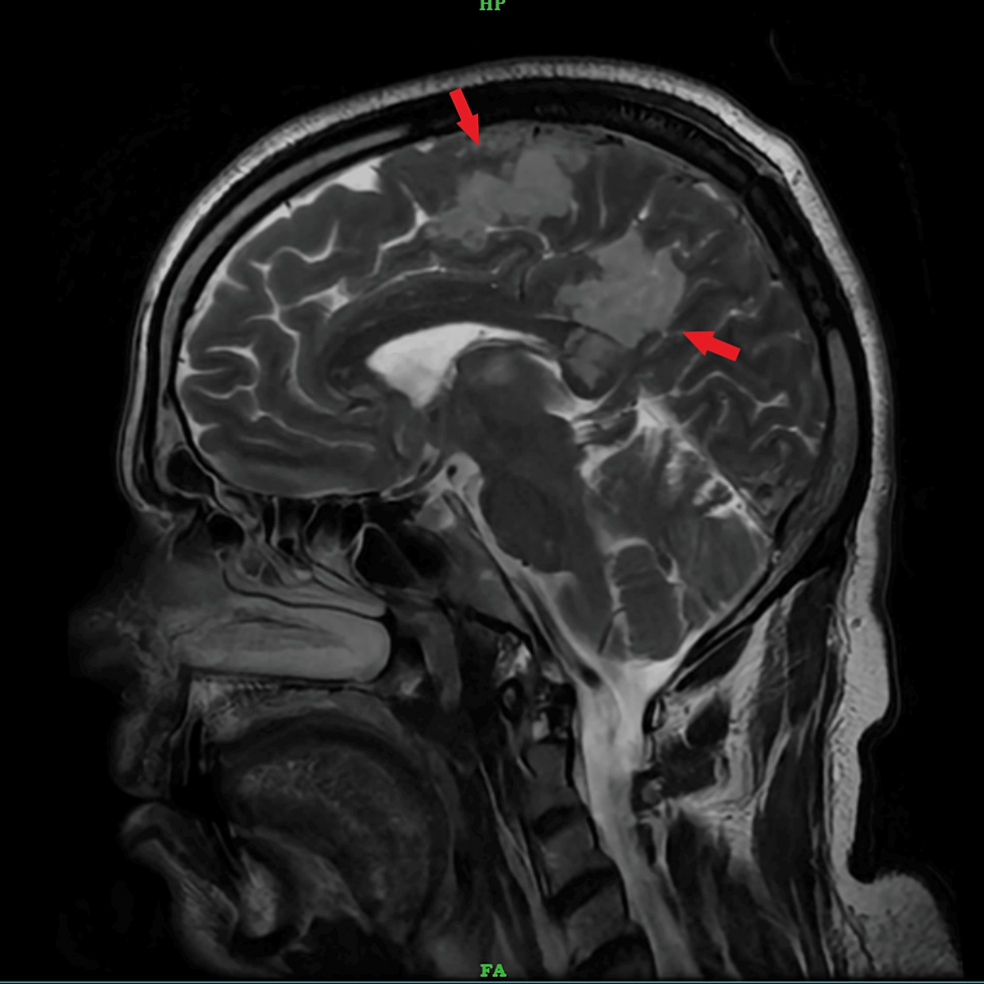
MRI brain T2-weighted sagittal section The red arrows indicate hyperintensities in the frontal lobe, cingulate gyrus, and splenium of the corpus callosum.

**Figure 3:**
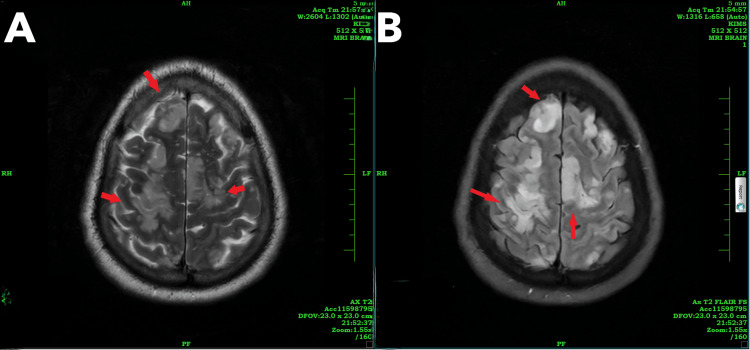
(A) T2-weighted MRI brain and (B) FLAIR sequence The red arrows highlight hyperintensities in the bilateral frontal cerebral hemispheres.

**Figure 4:**
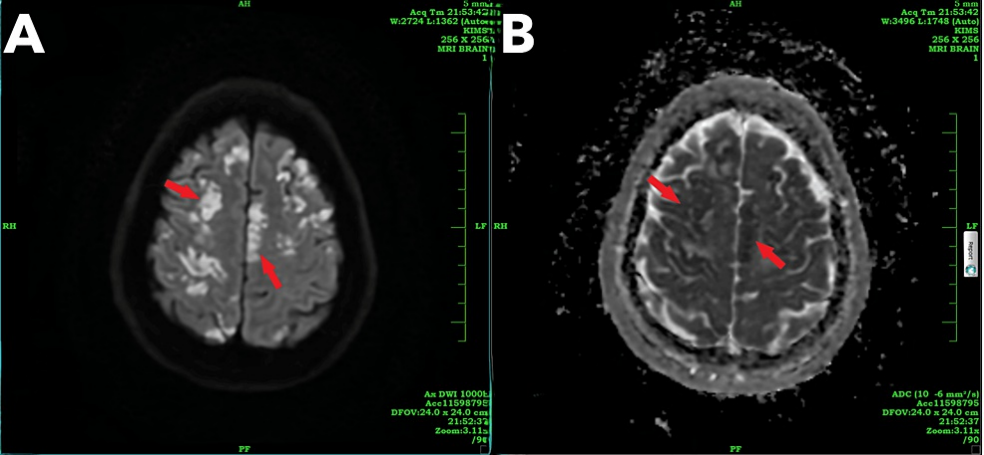
MRI brain showing (A) DWI and (B) ADC The red arrows indicate multiple ill-defined areas of hyperintensity on (A) DWI with corresponding hypointensity on (B) ADC, suggesting restricted diffusion noted in the bilateral frontoparietal lobes. ADC, apparent diffusion coefficient; DWI, diffusion-weighted imaging

After two days, the patient’s sensorium improved. Upon examination, the patient was conscious, oriented, and had UMN quadriparesis with bilateral plantar extensor response, Grade 3 deep tendon reflexes, and power of Grade 1/5 in all four limbs, without sensory symptoms. A contrast-enhanced MRI of the brain showed multiple areas of T2/FLAIR hyperintensities with diffuse restriction noted in the bilateral cerebral hemispheres, thalami, splenium of the corpus callosum, and bilateral cerebellar hemispheres, suggestive of acute infarction (Figures 2, 3). Multiple ill-defined areas of hyperintensity on diffusion-weighted imaging with corresponding hypointensity on apparent diffusion coefficient, indicating restricted diffusion, are noted in the bilateral frontoparietal lobes, suggestive of cytotoxic edema (Figure 4).

The patient received a diagnosis of an acute embolic stroke caused by hydrogen peroxide ingestion. Following this diagnosis, hyperbaric oxygen therapy was administered for five days, resulting in subsequent improvement in motor power to Grade 4/5 during the hospitalization period.

## Discussion

Hydrogen peroxide toxicity traces its origins back to the 1950s, when Ohm and Ciuti pioneered colonic irrigation using hydrogen peroxide to address meconium ileus [4]. However, by 1967, the perilous consequences of this practice had become apparent, prompting its discontinuation due to its potential lethality [5,6]. Presently, incidents of hydrogen peroxide toxicity primarily arise from accidental exposures, resulting in minor corneal defects, enteritis, and respiratory arrest [7-9].

Hydrogen peroxide ingestion can lead to various systemic complications, including neurologic sequelae such as acute ischemic stroke. In this case, the patient’s presentation with altered sensorium and subsequent development of UMN quadriparesis and hypoxemia highlights the potential for significant central nervous system and respiratory system involvement following hydrogen peroxide ingestion.

The pathophysiology of neurological injury in hydrogen peroxide ingestion is thought to involve multiple mechanisms, including direct toxicity, gas embolization, and the induction of systemic oxidative stress leading to endothelial dysfunction and thrombosis. The toxic effects of hydrogen peroxide are primarily due to its oxidizing properties and the liberation of oxygen gas upon decomposition. Ingesting a few milliliters of 35% hydrogen peroxide can produce significant amounts of oxygen. Under standard temperature and pressure, 30 mL of 35% hydrogen peroxide can release nearly 3.5 liters of oxygen [10]. The amount of oxygen gas released from the consumption of 50-60 mL of 50% w/w hydrogen peroxide is expected to be significant.

The release of oxygen gas can precipitate gas embolism, posing a significant systemic risk with the potential involvement of major vessels such as the vena cava, pulmonary arterial tree, and right heart chambers [10-13]. This may result in vascular obstruction, impeding blood flow, ischemia by diminishing oxygen supply to tissues, and organ damage, particularly affecting vital organs such as the brain, heart, and lungs. The multifocal nature of the ischemic lesions observed on imaging suggests widespread microvascular injury, likely secondary to embolic phenomena or small vessel thrombosis.

The case described here strongly indicates that the neurological injury is primarily attributed to gas embolization affecting the cerebral vasculature and pulmonary vasculature, which was evidenced clinically and by neuroimaging, which showed multiple areas of T2/FLAIR hyperintensities showing diffuse restriction noted in the bilateral cerebral hemisphere, thalami, splenium of the corpus callosum, and bilateral cerebellar hemisphere, suggestive of acute infarct, and a saddle-shaped thrombus in the main pulmonary trunk extending into both pulmonary arteries, suggestive of pulmonary embolism.

The initial management of hydrogen peroxide poisoning involves assessing the patient’s condition and ensuring their airway, breathing, and circulation are stable. Immediate attention is given to removing any remaining hydrogen peroxide from the mouth through rinsing and avoiding vomiting to prevent airway damage. Dilution therapy follows, with the administration of water or milk to reduce the concentration of hydrogen peroxide in the stomach. Gastrointestinal decontamination methods like gastric lavage or activated charcoal may be considered in specific cases. Supportive care, including oxygen therapy, intravenous fluids, and pain management, is provided as needed [14-16].

In severe cases involving oxygen gas embolism or neurological complications, hyperbaric oxygen therapy may be considered to improve outcomes. Following ingestion, immediate emergency measures may involve briefly positioning the patient in Trendelenburg with a left lateral decubitus tilt to facilitate the retrograde flow of bubbles from cerebral arteries. Additional therapies may include administering 100% oxygen, providing fluid support for tissue perfusion maintenance, and prescribing antiplatelet medication such as aspirin. Even if hyperbaric therapy is not readily available, delayed treatment can still offer benefits up to 30 hours after AGE symptom onset [10]. While spontaneous recovery from AGE is rare, previous cases have demonstrated persistent clinical and neuroradiologic abnormalities, highlighting the need for intervention. Hyperbaric oxygen therapy has shown efficacy in treating gas embolism from various causes, potentially improving tissue oxygenation in the ischemic penumbra of acute ischemic stroke.

## Conclusions

Monitoring and follow-up are integral parts of managing hydrogen peroxide poisoning. Continuous monitoring of vital signs, including heart rate, blood pressure, respiratory rate, and oxygen saturation, allows for timely intervention if there are any changes in the patient’s condition. Laboratory tests such as blood gas analysis, a complete blood count, and a metabolic panel help assess the patient’s overall health and response to treatment. Additionally, imaging studies such as a chest X-ray or CT scan may be performed to evaluate for gas embolism or pulmonary complications.

The prevention of hydrogen peroxide poisoning involves various measures aimed at reducing the risk of accidental ingestion. Proper storage of hydrogen peroxide out of reach of children and clear labeling can help prevent unintentional exposures. Education of family members about the dangers of ingesting household chemicals is essential in promoting awareness and safety. For those handling industrial concentrations of hydrogen peroxide, using appropriate personal protective equipment is crucial to minimize the risk of exposure and poisoning.
